# Measuring Situation Awareness: A Meta-Review Across Domains

**DOI:** 10.1177/00187208251412110

**Published:** 2026-01-17

**Authors:** Laura Louise Moens, Sinéad Lydon, Sara Cucurachi, Paul O’Connor, Thomas Christian Sauter, Gian-Andri Töndury, Tanja Manser

**Affiliations:** 1 University of Applied Sciences and Arts Northwestern Switzerland, Switzerland; 2 University of Bern Graduate School for Health Sciences, Switzerland; 3 8799 University of Galway, Ireland; 4 27252 Inselspital University Hospital Bern, Switzerland

**Keywords:** situation awareness, physiological measurement, psychometrics, team situation awareness, measures, vigilance (sustained attention)

## Abstract

**Objective:**

To identify and interpret evidence from systematic reviews of Situation Awareness (SA) measurement across domains, focussing on measures’ psychometric properties, and provide practical implications for SA measurement.

**Background:**

Several systematic reviews have been published on SA measurement, often focussing on specific measurement tools, domains, or psychometric properties. This creates a challenge for understanding the evidence supporting SA measures and for establishing best practice in SA measurement.

**Method:**

Five electronic databases were searched up to February 2025. The meta-review was prospectively registered (PROSPERO registration number: CRD42024521458). Reviews were included if they were systematic and focused on SA measurement. Data were extracted on the review characteristics and the SA measurement tools identified, including their psychometric properties. Studies were assessed using the Critical Appraisal Skills Programme checklist for systematic reviews.

**Results:**

Fourteen reviews, capturing over 477 unique primary studies, were included in this meta-review. In total, 38 distinct SA measurement tools were identified and subdivided into four categories of SA measurement: self-ratings, observer ratings, probing techniques, and physiological metrics. Psychometric evidence was limited for most tools. Probing techniques, especially the Situation Awareness Global Assessment Technique (SAGAT), showed the most extensive validity evidence but were associated with usability concerns.

**Conclusion:**

The application of SAGAT may be recommended as best practice currently, while other tools offer complementary strengths for specific contexts.

**Application:**

This synthesis provides guidance on best practice for SA measurement based on measurement purpose and context of use, balancing methodological rigour with feasibility to enhance SA measurement across diverse operational environments.

## Introduction

Human Factors research has emphasised the importance of Situation Awareness (SA) for effective performance in high-risk domains (e.g., Endsley, 1995b; Schulz et al., 2016). Reviews across diverse domains have examined and clarified the relevance of SA concepts and theories, consistently identifying Endsley’s (1995b) three-level model of SA as foundational to understanding SA (e.g., Meireles et al., 2018; Walshe et al., 2021). This model defines SA as the perception of information in the environment (SA Level 1), the comprehension of this information (SA Level 2), and the projection of future changes in the environment (SA Level 3; Endsley, 1995). Practitioners in various domains and researchers from disciplines beyond Human Factors are becoming increasingly interested in SA (Lopes et al., 2024; Willmer, 2017), often with a focus on assessing the impact of training interventions and digital tools on SA (e.g., Alqarrain et al., 2023; Brown et al., 2024). This increased interest is not solely attributable to the growing awareness of SA’s crucial role in safety but also relates to emerging technologies. Technologies such as augmented reality (AR) applications and artificial intelligence (AI) assistants have the potential to influence SA in both positive and negative ways. For instance, AI- and automation-driven displays can enhance SA when they provide transparent and comprehensible information (Endsley, 2023; van de Merwe et al., 2024), while AR systems can improve users’ SA when they are appropriately designed (Woodward & Ruiz, 2023). To assess how SA is impacted, valid, reliable, and usable SA measurement tools are required.

### Theoretical Foundations of Situation Awareness

Although Endsley’s (1995b) three-level model remains the most influential theoretical framework of SA, researchers have long noted that SA is a theoretically challenged construct, lacking a universally accepted operational definition and exhibiting ill-defined conceptual boundaries (Meireles et al., 2018; Sarter & Woods, 1991, 2017; Walshe et al., 2021). Sarter and Woods (1995) suggested that SA is “just a label for a variety of cognitive processing activities” in complex environments (p. 16). Other critics have argued that Endsley’s (1995b) levels of perception, comprehension, and projection oversimplify the intertwined and adaptive processes involved in maintaining SA (Dekker, 2015; Meireles et al., 2018; Stanton et al., 2010). These theoretical challenges have prompted the development of alternative perspectives on SA. One influential alternative is the constructivist perceptual cycle model (Smith & Hancock, 1995), which portrays SA not as static levels of knowledge but as part of a continuous sense-making loop. In this view, individuals’ expectations (mental models) guide what information they seek in the environment, and incoming information in turn updates those mental models, forming an ongoing cycle of perception and action.

Beyond the individual, collaborative approaches to SA have been introduced to emphasise that effective SA depends on a shared mental model or aligned understanding of the situation (Meireles et al., 2018; Salas et al., 1995; Walshe et al., 2021). The SA of groups has been conceptualised in two ways: as *team SA*, referring to the combined awareness of all team members, and *shared SA*, referring to the overlap in individual SA among team members (Ofte & Katsikas, 2023). Other researchers promoted a system approach, arguing against viewing SA as concentrated within individuals’ cognition (Bergström et al., 2011; Dekker, 2015; Flach, 1995; Hutchins, 1995; Salmon et al., 2015, 2017; Stanton et al., 2010). This distributed SA perspective frames SA as an emergent property of the socio-technical system comprising people, tools, and interfaces, residing in the interactions between human and non-human agents.

Taken together, this theoretical fragmentation illustrates that what constitutes SA depends on the context of use and unit of analysis (Walshe et al., 2021). Consequently, SA should be regarded as a context-dependent meta-construct rather than a singular, precisely defined phenomenon (Sarter & Woods, 1995), and this ambiguity has direct implications for the measurement of SA.

### Approaches to Situation Awareness Measurement

Conceptual diversity and ambiguity surrounding SA have led researchers to develop a range of measurement approaches that reflect differing interpretations of the construct and how it should be operationalised. One approach is to measure the cognitive *processes* that underlie SA (Endsley, 2021). Researchers have sought to measure these processes using, for example, communications analysis (e.g., Bolstad et al., 2007), verbal protocol analysis (e.g., Rose et al., 2019; Sullivan & Blackman, 1991; Walker et al., 2008), positional and movement acceleration data (e.g., Patil et al., 2023), EEG recording (e.g., De Winter et al., 2019; Sebastiani et al., 2020), and eye-tracking data (e.g., Anbro et al., 2020; De Winter et al., 2019; Zhang et al., 2023). Alternatively, researchers have focused on the *outcomes* of SA by measuring, for example, response time and errors (Endsley, 2021). In addition, researchers have sought to assess a person’s SA *directly* using subjective assessments such as self-ratings (e.g., the Situation Awareness Rating Technique) or observer ratings (e.g., the Situation Awareness Behavioural Rating System), and probe-based assessments in which individuals are asked questions about the situation that can be answered correctly or incorrectly (e.g., the Situation Awareness Global Assessment Technique; Endsley, 2020). Some of these direct assessments have been adapted for team-level application, in which individual scores are aggregated to represent overall team SA.

### Challenges in Situation Awareness Measurement

Each method of measuring SA has distinct advantages and limitations. Research on the psychometric properties of SA measurement tools has yielded mixed findings (e.g., Endsley, 2021; De Winter et al., 2019; Lau et al., 2014; Morgan et al., 2015). Measurement approaches can vary widely in terms of measurement sensitivity, ease of use, accuracy, predictive validity, susceptibility to error and bias, consistency, and intrusiveness.

In recent years, numerous reviews have been published on SA measurement, often focussing on specific measurement tools or domains of application (e.g., Arias-Portela et al., 2024; Endsley, 2021; Ghaderi et al., 2023). Consequently, the evidence on characteristics of existing SA measures and their psychometric properties is dispersed across different operational domains and research communities. The large number of reviews and heterogeneity in the underlying research questions and methodological approaches make it difficult to interpret the findings of those reviews and use them to decide which form of SA measurement to use (Gates et al., 2022). At the same time, measuring SA is becoming increasingly important and challenging in the context of emerging AI, automation, and AR technologies. Accordingly, the Human Factors community has a responsibility to provide guidance on best practice in SA measurement approaches for all researchers and practitioners concerned with measuring and enhancing SA.

### Review Objectives

We aim to generate best practice recommendations for SA measurement by identifying and interpreting evidence from systematic reviews on SA measures across domains using a meta-review approach, focussing on measures’ psychometric properties. Our review questions are:

(1) How is situation awareness measured in different contexts?

We consider the context of use in terms of *population* (e.g., pilots or healthcare professionals), *unit of measurement* (e.g., individuals or groups), and *setting* (e.g., simulation or real-life).

(2) What is the psychometric evidence of existing situation awareness measurement approaches and tools?

We consider all psychometric properties including *validity, reliability,* and *usability* (Asunta et al., 2019).

## Method

A review protocol was registered with the International Prospective Register of Systematic Reviews (PROSPERO; registration number: CRD42024521458) in March 2024. The review is reported in accordance with the Preferred Reporting Items for Overviews of Reviews (PRIOR) checklist (Gates et al., 2022).

### Review Design

Meta-reviews are referred to in various ways (e.g., overview of reviews, review of reviews, and umbrella review) and serve to gather, assess, and synthesise evidence from multiple systematic reviews on a specific topic (Gates et al., 2022). A meta-review methodology is particularly suitable for application to topics that have been extensively researched or reviewed. Meta-reviews adopt a systematic methodology. To ensure that this review was of a high standard, best practice in the conduct of meta-reviews was followed (Cant et al., 2022; Fusar-Poli & Radua, 2018).

### Search Strategy

A comprehensive search strategy was developed with the support of a research librarian (GT) and adapted for individual electronic databases. The search strategy (see Supplemental Material) comprised a combination of subject headings and free-text keywords relating to SA (e.g., ‘situation awareness’), measurement (e.g., ‘assessment’), and systematic review (e.g., ‘critical review’). Search terms were informed by publications on SA measurement (Cooper et al., 2013; Endsley, 2020, 2021; Ghaderi et al., 2023; Zhang et al., 2023), other meta-reviews (e.g., O’Connor et al., 2021), and validated peer-reviewed search blocks designed for meta-reviews (Liquitay et al., 2023; YOPL, 2018).

### Information Sources

Five databases (PsycINFO, Medline, Scopus, Web of Sciences, and Cochrane Database of Systematic Reviews) were initially searched in March 2024, and searches were last updated in February 2025. No search limits were applied. Following electronic searches, cited references (through backward citation chasing) and citing references (through forward citation chasing) were examined for all the reviews deemed eligible for inclusion as recommended by Hinde and Spackman (2015) and Papaioannou et al. (2010). Scopus, Web of Science, Citation Chaser, and Google Scholar were used to support these processes.

### Eligibility Criteria

Peer-reviewed papers were included if they were:

• available in English or Italian, reflecting the language expertise of the review team;
• systematic reviews, defined as containing a method section describing a systematic procedure for searching and selecting articles, intending to be exhaustive, and providing a visual and/or textual description of the screening process;
• focused on SA measurement.

### Selection Process

All database returns were exported to the web-based software Rayyan (Rayyan: Intelligent Systematic Review, 2021) and duplicates were removed. After deduplication, LM and SL piloted the eligibility criteria on a random sample of 10 reviews. Subsequently, LM screened the title and abstract of all returns and excluded studies that did not meet the eligibility criteria. The full texts of the remaining studies were screened and a final decision on inclusion was made. Any uncertainties were resolved through consulting with the wider research team (PC, SL, and TM).

### Data Extraction

Table 1 provides a full description of the data extraction variables for this review. Data were extracted by two authors (LM and SC) independently using a pre-formatted data extraction table, which was piloted by LM on two randomly selected articles and reviewed by the full research team. Any differences in data extracted were resolved through discussion.

**Table 1.** Data Extraction Variables Description and Coding.

Variables^a^		Descriptions	Coding
Author(s)		Last names of author(s) of the review	N/A
Publication year		The year in which the review was published	N/A
Review aim(s)		The research questions, aims, and/or hypotheses of the review	N/A
Period covered		The publication period covered by the included primary studies	N/A
Number of included studies		The number of primary studies included in the review	N/A
Quality assessment		The assessment tool used by the author(s) to evaluate the quality of the included studies, and the score and interpretation of the quality of the included studies	N/A
Measurement tools	Name	The name of the tool discussed in the review	N/A
	Description	A short description of the tool as reported by the author(s)	N/A
	Measurement type	The type(s) of measure used by the author(s) to categorise the tool, and the category to which each tool was assigned (only relevant if the authors categorised the tool)	Classified through inductive content analysis (Elo & Kyngäs, 2008) into the following categories: self-ratings, observer ratings, probing techniques, and physiological metrics
	SA level(s)	The SA level that the tool was used to measure	Classified into the following categories: Level 1, Level 2, and Level 3 as per Endsley (1995b)
	Administration	When and how often the measurement was administered	Classified into the following categories: continuous, intermittent, and post-hoc
	Population	The population in which the tool was used	N/A
	Unit of measurement	The unit of measurement to which the tool was applied	Classified into the following categories: individual, group, and system as per Ofte and Katsikas (2023)
	Setting	The setting in which the tool was used	Classified into the following categories: simulation or real-life setting
	Psychometric properties	The framework used by the author(s) to organise psychometrics-related evidence for the tool and/or the tool’s psychometric properties that were reviewed (only relevant if the authors categorised the psychometric evidence) All the information reported regarding the tool’s psychometric properties	Classified into the following categories: validity, reliability, and usability

N/A = not applicable. Variables that were not coded in this study are indicated by N/A. SA = situation awareness.

^a^These variables were informed by a meta-review with a similar research aim and method (i.e., O’Connor et al., 2021).

### Critical Appraisal

The Critical Appraisal Skills Programme (CASP) tool for systematic reviews was used to assess the methodological quality of the final set of included review papers. This tool has been widely applied in meta-reviews to interpret the relative strength of evidence and reporting across included systematic reviews (e.g., King et al., 2024; O’Connor et al., 2021; Reis et al., 2022). The CASP checklist offers clear guidance on applying ten items concerning (a) validity of the review results, (b) description of results, and (c) how the findings inform improvements in practice (CASP Checklists - Critical Appraisal Skills Programme, nd). Three items were not applied in our appraisal as they are only relevant for interventions: (7) “How precise are the results?”; (8) “Can the results be applied to the local population?”; (10) “Are the benefits worth the harms and costs?” CASP items require a response of ‘yes’ (=1 points), ‘no’ (=0 points), or ‘can’t tell’ (=0 points), returning a total score out of 7. The CASP was applied to the included reviews by LM and SC independently, and disagreements were resolved through discussion until consensus was reached.

### Data Synthesis

Elements of the data extracted from reviews were coded via deductive content analysis (Elo & Kyngäs, 2008) and are described in Table 1. The tools’ measurement type was coded through inductive content analysis (Elo & Kyngäs, 2008) because there is not one established categorisation for SA measurement tools that is used consistently across the literature.

Narrative synthesis, including a summary in textual and tabular form, was used to support the synthesis of the data extracted from the included reviews. Narrative synthesis is recognised as an excellent method for questions that are more suited to a qualitative consideration (Popay et al., 2006).

## Results

### Overview of Situation Awareness Measurement Reviews

**Figure 1.**
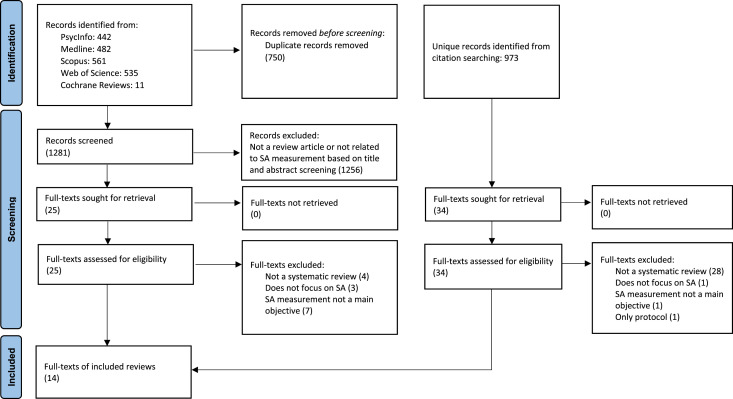
Flow diagram of the screening process reported according to the Preferred Reporting Items for Systematic reviews and Meta-Analyses (PRISMA) guidelines. *Note.* SA = situation awareness.

A total of 2254 articles were retrieved and screened, with 14 systematic reviews ultimately included (see Figure 1). Although Italian-language publications were included in the initial search strategy to ensure more comprehensive coverage, all reviews ultimately meeting the inclusion criteria were published in English. The included reviews contained at least 477 primary studies, of which 49 (±10%) appeared in more than one systematic review. Cooper et al. (2013) and Meireles et al. (2018) did not clearly report references to primary studies, so these are not reflected in the total count.

The included reviews were published between 2013 and 2024, primarily after 2020, and contained studies conducted between 1980 and 2024. In six reviews, SA measurement was the sole objective, whereas in eight reviews it was one of the main objectives (see Table 2). Other objectives were to identify theoretical foundations and definitions of SA and factors associated with SA. For example, Avalos et al. (2021) reviewed factors influencing nurses’ SA in addition to SA measures. Ten reviews focused on one specific domain: healthcare (5), driving (2), construction (1), sports (1), and cybersecurity (1), while the remaining four reviews were not specific to a domain. Table 2 provides a summary of the included reviews.

**Table 2.** Summary of Included Systematic Reviews in Alphabetical Order.

Author(s) and Publication Year	Review aim(s)	Domain	Number of Primary Studies Included in the Review	Period Covered by the Primary Studies Included in the Review	Tools for Measuring SA Included in the Review
Arias-Portela et al. (2024)	Elucidate the discernible relationship between eye-tracking metrics and SA in the context of driving, as well as the principal eye-tracking devices used, experimental designs, and a discussion	Driving	41	2011–2023	Eye-Tracking Metrics
Avalos et al. (2021)	(1) systematically review empirical studies of nurses’ SA, (2) summarize and evaluate the primary factors influencing nurses’ SA, and (3) provide an overview of available methodologies to measure nurses’ SA in inpatient settings	Healthcare	16	2005–2020	Interviews, Observation, Situation Awareness Global Assessment Technique (SAGAT), Situation Awareness Rating Technique (SART), Video Studies
Cheng and Esmaeili (2024)	Summarise and discuss (1) the level of SA in the current studies, (2) techniques to measure SA’s components, and (3) factors contributing to SA in the construction industry	Construction	16 (of which 12 concerned SA measurement)	2014–2021	Eye-Tracking Metrics, SAGAT, SART, Situation Presence Assessment Method (SPAM)
Cooper et al. (2013)	Review and describe indirect and direct measures of SA that have been tested in acute care/emergency settings	Healthcare	14	Not reported (electronic databases and search engines were searched from 1980 to 2010)^a^	Anaesthetists’ Non-Technical Skills (ANTS) System, Oxford Non-Technical Skills (NOTECHS) Scale, Non-Technical Skills for Surgeons (NOTSS), SAGAT, Team Emergency Assessment Measure (TEAM)
Endsley (2020)	Address how objective and subjective measures of SA relate to each other	Various (including military, aviation, air traffic control, submarine, manufacturing, IT, emergency management, driving, train driving, and robotics)	37 studies (in 34 articles^b^)	1998–2017	Crew Awareness Rating Scale (CARS), Low-Event Task Subjective Situation Awareness (LETSSA), Mission Awareness Rating Scale (MARS), Quantitative Assessment of Situation Awareness (QUASA), Situation Awareness Behavioural Rating System (SABARS), SAGAT, SART, SPAM
Endsley (2021)	Examine the SAGAT and SPAM methods for objective measurement of SA and review and compare the success of these metrics	Various (including aviation, air traffic control, driving, military, healthcare, manufacturing, robotics, unmanned air vehicle control, firefighting, train driving, maritime, teleoperations, emergency management, weather forecasting, cybersecurity, and submarine)	243 (in 238 articles^b^)	1988–2020	QUASA, Situation Awareness Control Room Inventory (SACRI), SAGAT, SALSA, Situation Awareness Verification and Analysis Tool (SAVANT), SPAM
Ghaderi et al. (2023)	Critically evaluate and summarise the quality of measurement properties in instruments used for measuring SA in healthcare professionals using the COSMIN methodology	Healthcare	25	2003–2019	ANTS, Anaesthetic Nontechnical Skills for Anesthetic Practitioners System (ANTS-AP), Explicit Professional Oral Communication (EPOC) Tool, ICARS, Oxford NOTECHS I, Oxford NOTECHS II, NOTSS, Non-Technical Skills for Urological Surgeons (NoTSUS), Ottawa Global Rating Scale (GRS), Situation Awareness Assessment Tool, SAGAT, Scrub Practitioners’ List of Intraoperative Non-Technical Skills (SPLINTS), Team Resuscitation Situation Awareness Tool, Trauma Non-Technical Skills (T-NOTECHS) Tool, Team Situation Awareness Global Assessment Technique (TSAGAT)
Huffman et al. (2022)	Identify (1) the frameworks labelled as SA in a sporting context, (2) the methods used to directly assess SA in sports, and (3) the cognitive skills explicitly associated with SA in sports	Sports	10 (of which 2 concerned SA measurement)	2006–2012	Cognition Self-Assessment Tool (CSAT), Random Number Cognition Test (RANCT), SAGAT
Meireles et al. (2018)	Review how individual SA is conceptualised and measured across divergent operational domains when only expert populations are considered	Various (including driving, aviation, healthcare, military, nuclear power plant, sports, and offshore drilling)	54	Not reported (research papers within the period between 1995 and 2015 were considered for an initial screening)^a^	Freeze Probes, Online Probes, Performance Measures, Post-Trial Probes, Self-Rating, Verbal Protocol Analysis
Ofte and Katsikas (2023)	Review the current research on SA within security operations centres to analyse (1) what theoretical foundations of SA are used, (2) what levels of conceptualisation for SA are used, and (3) what techniques for measuring SA are used	Cybersecurity	55	2012–2021	Performance, Proxy, SABARS, SAGAT, SART, Task Analysis
Orique and Despins (2018)	Examine various instruments and techniques used to measure SA among nurses across academic and clinical settings	Healthcare	40	2005–2015	ANTS, ANTS-AP, Anticipation, Checklist Perception Survey, Emergency Nurses’ Non-Technical Skills (ENNTS), Global Assessment of Obstetric Team Performance (GAOTP), Human Factors Rating Scale-Modified for Nurses (HFRS-MN), Imperial Military Personnel Assessment Tool (IMPAcT), Laparoscopic Cholecystectomy Postprocedure Assessment, Ottawa GRS, Oxford NOTECHS, Oxford NOTECHS II, Patient Review Time, Revised NOTECHS, SAGAT, SafeTeam, SPLINTS, T-NOTECHS, TSAGAT, Wondrous Original Method for Battle Airmanship Testing in Complex Systems (WOMBAT-CS)
Priambodo et al. (2022)	Examine (1) the simulation-based education learning strategies used to improve SA in nursing students and (2) tools commonly used to measure SA in simulation-based education in nursing students	Healthcare	9	2010–2020	Focus Groups and Interviews, Performance-Based Situation Awareness Observation Schedule (PBSAOS), SAGAT, 2010), Self-Situational Awareness Assessment Questionnaire (SSAAQ)
Tan and Zhang (2024)	Review the influential SA factors examined in empirical research related to Level 3 automated vehicles (AV) takeovers and to construct a conceptual model of these SA factors, provide design guidelines to facilitate driver SA restoration during Level 3 AV takeovers, and review SA assessment techniques used in the empirical studies to offer guidance on selecting suitable methods for evaluating driver SA during Level 3 AV takeovers	Driving	34	2013–2022	Close-Ended Questions, Gaze Behaviour, Interview, Non-Driving-Related Tasks Performance, Open-Ended Question, Post-Takeover Performance, Real-Time Probes, SAGAT, SART
Zhang et al. (2023)	Review (1) SA physiological measures, (2) the consistency of these metrics across various studies (including different tasks, equipment, and domains), and (3) the observed relationship between indirect and direct measures	Various (including construction, aviation, robotics, offshore drilling, police, air traffic control, healthcare, and driving)	25	2001–2019	CARS, Cardiovascular Metrics, Electrodermal Activity (EDA), Electroencephalography (EEG) Metrics, Eye-Tracking Metrics, QUASA, Respiratory Rate, SABARS, SAGAT, SART, SPAM

*Note*. Research aims are reproduced verbatim from the included articles. SA = situation awareness.

^a^For two reviews, we could not infer the time period covered because the references of the included studies were not included. These two reviews do include information about the time period that was screened, which we reported between brackets.

^b^We counted fewer articles in the reference list than the number reported by the author. We assume that the author counted the studies presented within the included articles and that some articles contained multiple studies.

### Overview of Situation Awareness Measurement Tools

In total, 38 tools for measuring SA emerged from the analysis of the reviews. Table 3 details all identified SA measurement tools. In this section, we present the results on tool characteristics, including the SA level they address and their administration. We also introduce the tool categories derived through inductive content analysis. Finally, we describe the context in which the tools were used, such as the population, unit of measurement, and setting.

**Table 3.** Overview of Tools Used to Measure Situation Awareness.

Category of Measure	Tool Name	Tool Description	SA Level(s)	Administration	Population	Unit of Measurement	Setting	Reference Systematic Review(s)
Self-Ratings	Cognition Self-Assessment Tool (CSAT)	Self-rating tool containing questions related to the degree to which participants would be able to carry out specific cognitive tasks	Overall SA	NR	Athletes	NR	Simulation	(Huffman et al., 2022)
	Crew Awareness Rating Scale (CARS) and the related Mission Awareness Rating Scale (MARS)	NR	Level 1, 2, and 3 as well as overall SA	NR	Healthcare professionals, pilots, robot operators, train drivers	NR	Real-life and simulation	(Endsley, 2020; Zhang et al., 2023)
	Low-Event Task Subjective Situation Awareness (LETSSA)	Self-rating asking operators to indicate the degree to which they agree with statements that describe behaviours associated with SA in the domain	NR	NR	Train drivers	NR	Real-life and simulation	(Endsley, 2020)
	SafeTeam	Self-rating questionnaire consisting of two parts assessing attitudes towards behaviours and self-reported application of tactics that optimise SA.	NR	NR	Healthcare professionals	NR	NR	(Orique & Despins, 2018)
	Self-Situational Awareness Assessment Questionnaire (SSAAQ)	Self-rating consists of three categories: the perception of awareness, the SA process of awareness, and SA skills	NR	NR	Healthcare professionals	NR	Simulation	(Priambodo et al., 2022)
	Situation Awareness Rating Technique (SART) and Team-Situation Awareness Rating Technology (T-SART)^a^	Self-rating approach administered on 7-point bipolar rating scales. Operators provide three global ratings of the basic dimensions (Demand, Supply, and Understanding), one overall SA rating (‘How good is your awareness of the situation?’), and 10 ratings of the elements composing the basic dimension (situation familiarity, attentional focus, information quantity and quality, situation instability, attention concentration, situation complexity, situation variability, arousal, and spare mental capacity). The technique then combines all 14 components for a final SART score	Level 1, 2, and overall SA	Post-hoc	Air traffic controllers, construction workers, drivers, emergency management team members, healthcare professionals, military personnel, offshore drilling operators, pilots, oil refinery operators, robot operators, security operations centre operators, submariners	Individual, team, and system	Real-life and simulation	(Avalos et al., 2021; Cheng & Esmaeili, 2024; Endsley, 2020; Ofte & Katsikas, 2023; Tan & Zhang, 2024; Zhang et al., 2023)
Observer Ratings	Anaesthetists’ Non-Technical Skills (ANTS) System	Observational 4-point rating scale designed to measure anaesthetists’ NTS in five categories: task management, team working, decision making, and SA. SA is divided into three elements: gathering information, recognising and understanding, and anticipating	Levels 1, 2, and 3	NR	Healthcare professionals	NR	Simulation	(Cooper et al., 2013; Ghaderi et al., 2023; Orique & Despins, 2018)
	Anaesthetic Nontechnical Skills for Anesthetic Practitioners (ANTS-AP) System	Observational 5-point rating scale designed to measure anaesthetic practitioners’ NTS in three categories (SA, teamwork, and communication) and nine elements	NR	NR	Healthcare professionals	NR	Simulation	(Ghaderi et al., 2023; Orique & Despins, 2018)
	Emergency Nurses’ Non-Technical Skills (ENNTS)	Adapted from ANTS, ENNTS was designed to evaluate NTS among emergency nurses. ENNTS is comprised of 12 items divided into four categories (communication, task management, SA, and decision making) scored on a 3-point Likert scale	Levels 1, 2, and 3	NR	Healthcare professionals	NR	NR	(Orique & Despins, 2018)
	Explicit Professional Oral Communication (EPOC) Tool	Not reported	NR	NR	Healthcare professionals	NR	Real-life	(Ghaderi et al., 2023)
	Global Assessment of Obstetric Team Performance (GAOTP)	Observational 5-point rating scale designed to assess obstetrical team performance in six categories: Communication, task/case management, teamwork, SA, communication, and room environment	NR	NR	Healthcare professionals	NR	NR	(Orique & Despins, 2018)
	Human Factors Rating Scale-Modified for Nurses (HFRS-MN)	Adapted from NOTECHS, the HFRS-MN was designed to evaluate NTS among surgical nurses on five teamwork dimensions (communication and interaction, vigilance/SA, team skills, leadership and management, decision making) scored on a 6-point Likert scale	Overall SA	NR	Healthcare professionals	NR	Simulation	(Orique & Despins, 2018)
	Imperial Military Personnel Assessment Tool (IMPAcT)	Observational 7-point rating scale designed to measure military hospital employees’ skills in four primary categories (nontechnical/crisis management skills, hostile environments, trauma care, and transfer of learning)	NR	NR	Healthcare professionals	NR	Simulation	(Orique & Despins, 2018)
	Interpersonal and Cognitive Assessment for Robotic Surgery (ICARS)	Observational 5-point rating scale with four subscales and 28 items	NR	NR	Healthcare professionals	NR	Simulation	(Ghaderi et al., 2023)
	Non-Technical Skills for Surgeons (NOTSS)	Observational 4-point rating scale designed to measure surgeons’ NTS in 5 categories: SA, decision making, leadership, communication and teamwork, and task management	Levels 1, 2, and 3	NR	Healthcare professionals	NR	NR	(Cooper et al., 2013; Ghaderi et al., 2023)
	Not-Technical Skills for Urological Surgeons (NoTSUS)	Observational 5-point rating scale based on the NOTSS, designed to measure urological surgeons’ NTS.	NR	NR	Healthcare professionals	NR	Simulation	(Ghaderi et al., 2023)
	Ottawa Global Rating Scale (GRS)	Observational 7-point rating scale designed to evaluate crew resource management performance in five categories: leadership, problem-solving, SA, resource utilization, and communication	Overall SA	NR	Healthcare professionals	NR	Simulation	(Ghaderi et al., 2023; Orique & Despins, 2018)
	Oxford Non-Technical Skills (NOTECHS) Scale	Observational 4-point rating scale designed to measure NTS in operating department assistants, scrub nurses, anaesthetists, and surgeons in four categories: leadership and management, teamwork and cooperation, problem-solving and cooperation, and SA. SA is measured in slightly different ways for each professional group	Overall SA	NR	Healthcare professionals	Team	Real-life and simulation	(Cooper et al., 2013; Ghaderi et al., 2023; Orique & Despins, 2018)
	Oxford Non-Technical Skills (NOTECHS) II Scale	Similar to the Oxford NOTECHS, the Oxford NOTECHS II consists of four categories but it is scored using an 8-point Likert scale	NR	NR	Healthcare professionals	Team	Real-life	(Ghaderi et al., 2023; Orique & Despins, 2018)
	Performance-Based Situation Awareness Observation Schedule (PBSAOS)	Consists of 54 items that measure the presence or absence of nursing students’ SA abilities	NR	NR	Healthcare professionals	Individual	Simulation	(Priambodo et al., 2022)
	Revised NOTECHS	A revised version of the Oxford NOTECHS developed to include communication and interaction using a 6-point Likert scale for scoring	NR	NR	Healthcare professionals	NR	NR	(Orique & Despins, 2018)
	Scrub Practitioners’ List of Intraoperative Non-Technical Skills (SPLINTS)	Observational 4-point rating scale designed to measure scrub practitioners’ NTS in three categories: SA, communication and teamwork, and task management	Overall SA	NR	Healthcare professionals	NR	Real-life	(Ghaderi et al., 2023; Orique & Despins, 2018)
	Situation Awareness assessment Tool	Observational checklist with 14 items divided over three subscales	NR	NR	Healthcare professionals	NR	Simulation	(Ghaderi et al., 2023)
	Situation Awareness Behavioural Rating System (SABARS)	Observational 5-point rating scale on 28 behaviours associated with good SA.	NR	NR	Military personnel, police, security operations centre operators	Team	Real-life and simulation	(Endsley, 2020; Ofte & Katsikas, 2023; Zhang et al., 2023)
	Team Emergency Assessment Measure (TEAM)	Observational 4-point rating scale designed to measure emergency team NTS on 11 items grouped under three categories: leadership, teamwork, and task management. A 12th item is included as a global ‘overall’ rating of team performance. TEAM includes two elements related to SA.	Levels 1 and 3	NR	Healthcare professionals	Team	NR	(Cooper et al., 2013)
	Team Resuscitation Situation Awareness Tool	Observational 5-point scale checklist with seven items measuring individual SA and summing these individual scores to get a team SA score	NR	NR	Healthcare professionals	Team	Simulation	(Ghaderi et al., 2023)
	Trauma Non-Technical Skills (T-NOTECHS) Tool	Observational 5-point rating scale designed to measure trauma teams’ NTS in 5 categories: leadership, cooperation and resource management, communication and interaction, assessment and decision making, and SA/coping with stress	NR	NR	Healthcare professionals	NR	Real-life and simulation	(Ghaderi et al., 2023; Orique & Despins, 2018)
Probing Techniques	Quantitative Assessment of Situation Awareness (QUASA)	A variant of SAGAT that uses true/false statements as probes and adds an assessment of confidence to each answer	NR	Intermittent	Military personnel, robot operators	Individual and team	NR	(Endsley, 2020, 2021; Zhang et al., 2023)
	SALSA	A variant of SAGAT that weighs each query type in computing an overall score and was created to measure SA of area controllers within the context of automation	NR	Intermittent	NR	Individual and team	NR	(Endsley, 2021)
	Situation Awareness Control Room Inventory (SACRI)	A variant of SAGAT that computes a sensitivity and bias score based on signal detection theory	NR	Intermittent	Drivers	Individual and team	NR	(Endsley, 2020, 2021)
	Situation Awareness Global Assessment Technique (SAGAT) an Team Situation Awareness Global Assessment Technique (TSAGAT)^a^	Scenarios are frozen at randomly selected times, and system displays are blanked while people quickly answer questions about their current perceptions of the situation. Queries can be provided verbally, via pencil and paper, or on a computer or tablet. SAGAT queries correspond to an individual’s SA requirements as determined based on an SA requirements analysis for a given domain and role. The answers to the queries are compared with ground truth, as collected from simulation computers or subject matter experts with perfect knowledge of the situation, to provide an objective assessment of SA accuracy. Using TSAGAT, team SA is calculated by summing each team member’s individual SAGAT score	Level 1, 2, and 3 as well as overall SA	Intermittent	Air traffic controllers, athletes, construction workers, drivers, emergency management team members, firefighters, health care professionals, military personnel, nuclear power plant control room operators, oil refinery operators, pilots, robot operators, security operations centre operators, submariners, train drivers, unmanned air vehicle controllers	Individual and team	Real-life and simulation	(Avalos et al., 2021; Cheng & Esmaeili, 2024; Cooper et al., 2013; Endsley, 2020, 2021; Ghaderi et al., 2023; Huffman et al., 2022; Ofte & Katsikas, 2023; Orique & Despins, 2018; Priambodo et al., 2022; Tan & Zhang, 2024; Zhang et al., 2023)
	Situation Awareness Verification and Analysis Tool (SAVANT)	A variant of SAGAT that provides a partial display to ask questions about missing information and includes an assessment of time to answer each question	NR	Intermittent	NR	Individual and team	NR	(Endsley, 2021)
	Situation Present Assessment Method (SPAM)	Real-time probe technique. SA probes are provided one at a time during a task with displays in view, and both the time to respond and the accuracy of responses are measured. The SPAM also provides a ready prompt before the probe that allows participants to defer answering until they are ready. SPAM probes are designed to assess participants’ knowledge of the past, present, and future. SA probes may be staged as an embedded probe, with a confederate playing the role of the questioner	Levels 1, 2, and 3	Intermittent	Air traffic controllers, construction workers, drivers, military personnel, pilots, process controllers, robot operators, submariners	Individual and team	Real-life and simulation	(Cheng & Esmaeili, 2024; Endsley, 2020, 2021; Tan & Zhang, 2024; Zhang et al., 2023)
Physiological Metrics	Cardiovascular Metrics	There are two main cardiovascular metrics. Heart rate (HR) measures heartbeats per minute, and average HR is expected to increase as SA increases. HRV is a measure of neurocardiac function that provides information about heart–brain interaction and the autonomic nervous system. HRV is expected to decrease as SA increases	NR	NR	Drivers, offshore drilling operators, pilots, police	NR	NR	(Zhang et al., 2023)
	Electrodermal Activity (EDA)	Not reported	NR	NR	Pilots	NR	NR	(Zhang et al., 2023)
	Electroencephalography (EEG) Metrics	One of the most standard methods of EEG analysis is frequency-domain analysis, which categorises EEG signals into different types of brain waves according to their frequency. Increased power at higher frequencies is associated with more alertness. Frequency bands that are associated with alertness and arousal are expected to be more active if SA is higher	Levels 1, 2, and 3	Continuous	Pilots	NR	NR	(Zhang et al., 2023)
	Eye-Tracking Metrics	Eye-tracking techniques measure an individual’s eye movements in two-dimensional space. In general, longer times in task-relevant areas of interest and more dispersed gaze patterns are taken as indications of better SA.	Level 1	Continuous	Air traffic controllers, construction workers, drivers, healthcare professionals, nuclear power plant control room operators, pilots, robot operators	NR	Real-life and simulation	(Arias-Portela et al., 2024; Cheng & Esmaeili, 2024; Tan & Zhang, 2024; Zhang et al., 2023)
	Respiratory Rate	Not reported	NR	NR	Pilots	NR	NR	(Zhang et al., 2023)

*Note*. NR = not reported. NTS = non-technical skills. SA = situation awareness.

^a^SART and T-SART are presented together because the only difference between SART and T-SAGAT is that SART scores of multiple team members are combined to get the T-SART score. The administration, rating scale, etc., of both tools are the same. This also applies to SAGAT and T-SAGAT.

#### SA Level

All reviews referred to Endsley’s (1995b) three-level definition of SA. Most tools have been used to measure all three SA levels or overall SA (see the ‘SA-Level(s)’ column in Table 3), while some, such as eye-tracking metrics, focused on SA level 1 (i.e., perception).

#### Tool Administration

Most reviews did not provide details on how the tools were administered. Among the reported approaches, intermittent administration was the most frequently reported one, compared to continuous and post-hoc administration (see the ‘Administration’ column in Table 3). Intermittent measurement was most commonly implemented using the Situation Awareness Global Assessment Technique (SAGAT) and related techniques. Post-hoc measurement has been conducted using the Situation Awareness Rating Technique (SART). For continuous SA measurement, physiological tools such as eye-tracking and electroencephalography (EEG) metrics have been used.

#### Type of Measure

**Figure 2.**
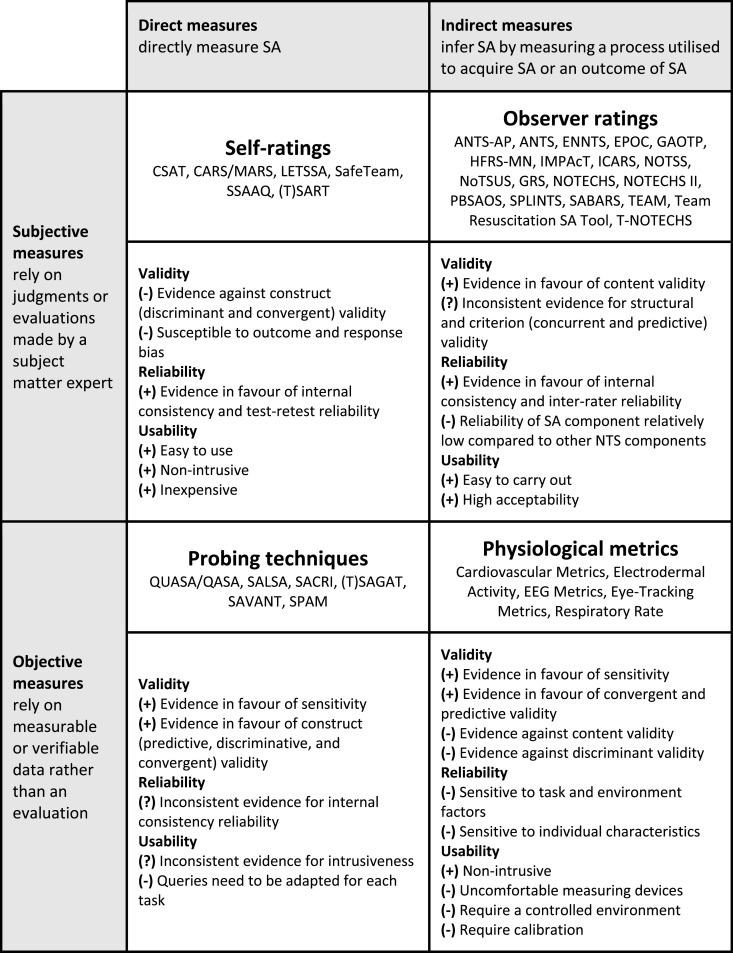
Classification matrix for situation awareness measures and associated psychometric evidence. *Note*. (+) indicates predominantly positive evidence, (−) indicates predominantly negative evidence, and (?) indicates inconsistent evidence (i.e., both positive and negative evidence). NTS = non-technical skills.

Some reviews distinguished between direct and indirect measures (e.g., Orique & Despins, 2018). Direct measures obtain SA data directly, whereas indirect measures infer SA based on a process used to acquire SA (e.g., visual attention) or an outcome of SA (e.g., reaction time). In addition, other reviews distinguished between subjective and objective SA measures (e.g., Endsley, 2020). Subjective measures require either the individual themselves or an external observer to evaluate SA, whereas objective measures rely on measurable data rather than personal evaluation. We combined these two dimensions to create a 2 × 2 matrix, with each cell representing a category of SA measures: self-ratings, observer ratings, probing techniques, and physiological metrics (Figure 2). We assigned all tools to one of these four categories. The psychometric evidence for tools within each category is summarised at category-level in Figure 2 and described in detail in the following section.

#### Population

Most of the tools identified in this review have been applied to measure the SA of healthcare professionals (*n* = 26; 68%), followed by pilots (*n* = 9; 24%) and drivers (*n* = 6; 16%) (see Table 3 ‘Population’). Observer ratings have been used specifically with healthcare professionals while other tools have been used across different population groups. Study populations included both trainees (e.g., Priambodo et al., 2022) and experts (e.g., Meireles et al., 2018).

#### Unit of Measurement

Most reviews did not specify the unit of measurement used for SA assessment. Individual and group SA were reportedly measured using probing techniques, such as SAGAT (see the ‘Unit of Measurement’ column in Table 3). Several observer rating tools were specifically designed for assessing group SA, including the Team Emergency Assessment Measure (TEAM). According to one review, system SA was measured using SART and proxy measures such as eye-tracking metrics.

#### Setting

Tools were predominantly used in simulation settings (*n* = 21; 84% of the tools for which the setting was reported) compared to real-life settings (*n* = 12; 48%) (see Table 3 ‘Setting’). Nine tools (36%) were reportedly used in both settings.

### Self-Ratings

Eleven out of 14 reviews discussed self-ratings (see Table 2). A description of all identified self-rating tools can be found in Table 3. Self-ratings are a post-trial approach where operators provide ratings about their SA on a Likert scale. The most reported self-rating tool is SART.

According to the systematic reviews, most self-ratings capture all three SA levels and/or overall SA (see Table 3). As can be seen from Table 3, self-ratings have been used in various populations, including healthcare professionals, pilots, construction workers, athletes, cybersecurity operators, and train drivers, in both simulation and real-life settings.

#### Validity of Self-Ratings

Some evidence raised questions about the validity of self-ratings. Available validity evidence for SA self-ratings came exclusively from Endsley (2020) who analysed how two objective measures (i.e., SAGAT and SPAM) relate to various self-rating methods across domains. The study highlighted a strong divergence between self-ratings and these probing techniques. More specifically, some of the primary studies found moderate correlations between SART and probe-based SA measures, but most studies reported no significant correlations. Similarly, inconsistent results were found for the convergent validity of the Crew Awareness Rating Scale (CARS) and the related Mission Awareness Rating Scale (MARS), and only one primary study in a train driving simulation showed a positive correlation between the Low-Event Task Subjective Situation Awareness (LETSSA) and SAGAT (i.e., Rose et al., 2018).

Endsley (2020) explains the divergence between self-ratings and probe-based measures of SA, arguing that the former reflect individuals’ confidence in their SA and workload rather than SA itself. This argument is supported by primary studies showing a significant positive correlation between SART and a scale for measuring task load (i.e., the NASA Task Load Index) and between subjective SA and confidence. In addition, SART answers can be biased by outcomes (Tan & Zhang, 2024). For example, individuals who achieve successful task performance may rate their SA more favourably, even if their objective SA was low, whereas those who perform poorly might underrate their SA. Moreover, a review from the cybersecurity domain emphasises that self-ratings are sensitive to response bias (Ofte & Katsikas, 2023). Self-ratings’ generally weak or non-existent correlation with SAGAT and SPAM, along with their association with other factors, suggests low concurrent and discriminant validity.

#### Reliability of Self-Ratings

Only little, but positive evidence was found for the reliability of SA self-ratings. While SART is the most popular self-rating tool, no evidence of reliability was reported for SART in the included reviews. Priambodo et al. (2022) found positive evidence of the internal consistency of the Self-Situational Awareness Assessment Questionnaire (SSAAQ) used in a sample of nursing students (Cronbach’s α = .76). Huffman et al. (2022) reported one study in the sports domain that suggests the Cognition Self-Assessment Tool (CSAT) had high test-retest reliability.

#### Usability of Self-Ratings

Self-ratings benefit from the ease of use in both administration and analysis, in addition to being inexpensive and non-intrusive (Cheng & Esmaeili, 2024). Self-ratings appear to be practical tools, as indicated by studies in the cybersecurity domain (Ofte & Katsikas, 2023), in automated driving experiments (Tan & Zhang, 2024), and in the construction industry (Cheng & Esmaeili, 2024).

### Observer Ratings

Eight of 14 reviews discussed observer ratings (Table 2). A description of all identified observer rating tools can be found in Table 3. Observer ratings are based on the observation of the behaviour of individuals or teams, usually by a trained subject matter expert, on pre-established items using a Likert scale. It is important to note that there are observer ratings specifically designed to assess SA and others used to assess non-technical skills (NTS), amongst which SA forms one category.

Like self-ratings, observer ratings have mostly been used to assess either all three levels of SA or the overall SA (see Table 3). As can be seen from Table 3, observer ratings have mainly been developed and used for assessing SA in healthcare professionals. Only the Situation Awareness Behavioral Rating System (SABARS) was reportedly not used with healthcare professionals. Instead, SABARS has been used to assess the SA of security operation centre operators, police, and (non-medical) military personnel.

#### Validity of Observer Ratings

From the included reviews, there was little evidence for the validity of observer ratings that exclusively assess SA. Endsley (2020) presented a study involving 14 infantry-domain participants, which found that SABARS is positively correlated with SAGAT. Ghaderi et al. (2023) analysed the content validity of the Team Resuscitation SA Tool and the SA Assessment Tool but found no convincing evidence in favour or against their validity.

Ghaderi et al. (2023) also evaluated the content validity of various NTS observer ratings, focussing on the SA component. For some observer ratings, such as the Non-Technical Skills for Surgeons (NOTSS), Anaesthetists’ Non-Technical Skills (ANTS) System, Anaesthetic Nontechnical Skills for Anesthetic Practitioners (ANTS-AP) System, Interpersonal and Cognitive Assessment for Robotic Surgery (ICARS), and Scrub Practitioners’ List of Intraoperative Non-Technical Skills (SPLINTS), the available evidence suggested that these tools covered key aspects of SA. However, for other instruments, such as the Not-Technical Skills for Urological Surgeons (NoTSUS) and Explicit Professional Oral Communication (EPOC) Tool, the evidence was weaker, making it unclear whether they fully captured the intended construct. Orique and Despins (2018) also reviewed various NTS observer ratings in healthcare settings and reported positive evidence for the content validity of the Imperial Military Personnel Assessment Tool (IMPAcT). TEAM was also reported to have a very high content validity by Cooper et al. (2013).

Structural validity, defined as the extent to which the dimensions of the construct assessed by the instrument align with the underlying theory, was rarely assessed according to Ghaderi et al. (2023). When it was examined, such as in the case of NOTSS, the results were inconsistent, raising concerns about whether the rating categories truly reflect distinct aspects of SA.

Criterion validity, which tests how well a measure aligns with an established ‘gold standard’ measure, was assessed in a few studies included in Ghaderi et al. (2023). However, most studies relied on similar rating tools rather than an objective benchmark. For example, NoTSUS scores were found to correlate strongly with those from NOTSS. Some studies also tested whether observer ratings correlated with expected outcomes. Although Orique and Despins (2018) included a primary study that showed a negative association between the Oxford Non-Technical Skills (NOTECHS) Scale and surgical errors, overall findings of predictive validity were inconsistent (Ghaderi et al., 2023), making it uncertain whether observer ratings genuinely measure SA as intended.

#### Reliability of Observer Ratings

The reliability of observer ratings has been assessed across multiple healthcare-focused reviews, revealing inconsistent results for different tools (Cooper et al., 2013; Ghaderi et al., 2023; Orique & Despins, 2018; Priambodo et al., 2022). Ghaderi et al. (2023) found moderate evidence suggesting sufficient internal consistency for the SA Assessment Tool and ICARS, with Cronbach’s alpha values higher than 0.70, while the available evidence for NOTSS was inconsistent. Orique and Despins (2018) reported that ANTS, ANTS-AP, EENTS, Human Factors Rating Scale-Modified for Nurses (HFRS-MN), IMPAcT, and NOTECHS demonstrated high reliability. In contrast, they observed more inconsistencies in the reliability for NOTECHS II, Trauma Non-Technical Skills (T-NOTECHS) Tool, Ottawa Global Rating Scale (GRS), and SPLINTS. Priambodo et al. (2022) reviewed the Performance-Based Situation Awareness Observation Schedule (PBSAOS) and found high interrater reliability.

The reliability of observer ratings seems to be lower for SA than for other NTS categories (Cooper et al., 2013). Cooper et al. (2013) reviewed NOTSS and reported high internal consistency and good interrater reliability for some categories, but poor reliability for SA. They reported similar results for a revised version of NOTECHS. In addition, they found a satisfactory internal consistency for TEAM and ANTS, but interrater reliability was not assessed for individual TEAM items and was lowest in the SA category for ANTS.

#### Usability of Observer Ratings

An advantage of observer ratings is that they are easy to carry out (Avalos et al., 2021; Orique & Despins, 2018). Orique and Despins (2018) found that the acceptability and usability of ANTS ranged between 78% and 100%.

### Probing Techniques

Twelve out of 14 reviews discussed probing techniques (see Table 2). A detailed description of all identified probing techniques can be found in Table 3. There are two main kinds of probing techniques: freeze probes and real-time probes. Freeze probes involve freezing a task at unpredictable time points and questioning individuals about their current perception, comprehension, and projection of the situation. The answers to the questions are compared with answers from experts with full knowledge of the situation to provide a verifiable assessment of SA accuracy. The most well-known freeze probe tool is the Situation Awareness Global Assessment Technique (SAGAT). Real-time probes are administered one at a time during task performance, measuring both the accuracy of responses and the time to respond. The most popular real-time probe tool is the Situation Present Assessment Method (SPAM). SPAM also offers the respondent the option to defer answering to the probe until it is suitable. Real-time probes can be integrated into the scenario, for example, by having a confederate posing the questions.

According to the systematic reviews, probing techniques have been used to measure each of the three SA levels and they have been applied both in simulation and in real-life settings (see Table 3). As can be seen from Table 3, real-time and freeze probes have been used to measure SA in pilots, construction workers, air traffic controllers, military personnel, drivers, submariners, robot operators, and process controllers. Freeze probes have also been used in other population groups, which can be found in Table 3.

#### Validity of Probing Techniques

Probing techniques, especially freeze probes, were the most often validated SA measure and were considered to have greater validity compared to, for example, observer ratings, due to their relatively objective nature (Cooper et al., 2013; Orique & Despins, 2018).

Two meta-analyses presented evidence for the validity of SAGAT across domains (Endsley, 2020, 2021). Endsley (2021) looked at SAGAT’s sensitivity to factors that are expected to influence SA, such as system and automation manipulations, as well as differences in expertise and operational concepts. Sensitivity was calculated as the percentage of primary studies in which SAGAT detected a theorised difference in SA between study conditions. A sensitivity score of 85.5% across 152 studies was found. Moreover, SAGAT’s sensitivity was independent of the domain and the experience of test subjects.

However, Endsley (2021) found that sensitivity was impacted by the way the measure had been administered. For example, some studies only measured SA at the end of trials, combined the scores of all queries, or administered only one or two freezes. When only considering the 64 studies that used SAGAT as intended (i.e., with scenario freezes, analysis by SA level or by query, and adequate sample size), overall sensitivity rose to 94%. SAGAT design is not only important for its sensitivity. SAGAT’s susceptibility to biases also depends on SAGAT design, including whether the queries are relevant, cover a wide range of SA requirements, and are administered at random times (Endsley, 2021; Tan & Zhang, 2024).

Furthermore, Endsley (2021) found evidence for high predictive validity of SAGAT. Moreover, Endsley (2021) found that *shared SA* calculated based on SAGAT scores, was predictive of overall team performance. By comparison, Huffman et al. (2022) looked at studies in the sports domain and found evidence that SAGAT scores were not a significant predictor of performance scores. According to Huffman et al. (2022), this might be the case because sports is a domain where physical qualities, in addition to SA, are very important for performance.

Besides sensitivity and predictive validity, Endsley (2021) also looked at the discriminant validity of SAGAT. More specifically, the meta-analysis focused on the relationship between SAGAT scores and working memory and found no support for such a relationship. Endsley (2020) analysed the correlation between SAGAT and subjective measures like SART and found no significant correlation across the included primary studies. According to Endsley (2020), the generally weak or non-existent correlation between both SA measures is due to the low validity of SART. In addition, Orique and Despins (2018) looked at convergent validity and found positive correlations between SAGAT and knowledge.

Considerably less evidence is available for variants of SAGAT. For the Situation Awareness Control Room Inventory (SACRI), Endsley (2021) found no correlation with SART and an overall sensitivity of 75% based on four studies. The Quantitative Assessment of Situation Awareness (QUASA), which assesses both the accuracy of answers as well as subjects’ SA confidence, was shown to correlate positively with both SAGAT and CARS in one primary study (Endsley, 2020). Another primary study found a negative correlation between QUASA *accuracy* and SART scores and a positive correlation between QUASA *confidence* and SART scores. In Endsley (2021), four of the six primary studies (67%) using QUASA found that it was sensitive. For SALSA, Endsley (2021) found a sensitivity of 100% and a predictiveness of 50% across two studies.

The SPAM has also been subjected to a thorough review (Endsley, 2020, 2021). Similar to SAGAT, SPAM appeared to be predictive of performance and team performance and did not show a significant correlation with SART (Endsley, 2021). However, SPAM had a lower sensitivity score than SAGAT, of only 64%.

Furthermore, there are concerns about SPAM’s discriminant validity due to correlations between SPAM scores and workload (Endsley, 2021). According to Endsley (2021), this could be due to the design of SPAM. When using SPAM, the simulation is not frozen, and relevant information is available on the displays, allowing for checking information in the environment before answering a query. Additionally, Endsley (2021) noted that SPAM may partly measure memory, as it includes questions about past events, but no empirical evidence for this conjecture is provided in the systematic review. Finally, Endsley (2021) argues that SPAM is sensitive to sampling bias because subjects can postpone queries until a moment when their workload is lower.

#### Reliability of Probing Techniques

We found inconsistent evidence for the reliability of probing techniques. Ghaderi et al. (2023) examined the reliability of SAGAT across five studies but evidence was reported in only two of them, which was positive but of low quality, due to factors such as indirect evidence. Priambodo et al. (2022) reported a Cronbach’s alpha ranging from .373 to .723 in a primary study that included nursing students. Additionally, no evidence has been reported for the reliability of SAGAT variants or real-time probes.

#### Usability of Probing Techniques

The evidence regarding the usability of probing techniques is inconsistent. SAGAT was found to be intrusive (Cheng & Esmaeili, 2024). Probing participants during freezes limits SAGAT’s suitability for real-life situations (Meireles et al., 2018) and scenario freezes may negatively affect performance and alter SA (Cooper et al., 2013). In contrast, other reviews demonstrated that SAGAT does not affect performance and is, therefore, non-intrusive (Endsley, 2021; Orique & Despins, 2018). Cheng and Esmaeili (2024) report a study from the construction domain that found SPAM to be less intrusive than SAGAT. In contrast, Endsley (2021) reported that six out of 15 (40%) of the studies that used SPAM showed a negative impact on performance or workload, while none of the 11 studies examining the effect of SAGAT found such a negative effect. In addition to intrusiveness, another potential usability issue is that developing queries requires in-depth task analysis, and queries cannot be reused as readily as the items of subjective rating tools (Ghaderi et al., 2023).

### Physiological Metrics

Four out of 14 reviews discussed physiological metrics (see Table 2). A description of all identified physiological metrics associated with SA can be found in Table 3. Physiological metrics-based tools use technology to measure key parameters of the human body, such as heart rate, eye movement, and brain activity, as indicators of SA. The most used physiological metric for SA measurement is eye tracking.

According to the included reviews, eye tracking is used to capture visual attention and is therefore useful for measuring level 1 SA, whereas EEG was used to measure all three SA levels (see Table 3). As can be seen in Table 3, eye tracking and EEG are both continuous measures. Physiological metrics have been used mostly with pilots, but eye tracking has also been applied to drivers, security operations centre operators, construction workers, air traffic controllers, healthcare professionals, robot operators, and nuclear power plant control room operators. Moreover, the reviews showed that eye tracking has been used to measure SA both in simulation and real-life settings.

#### Validity of Physiological Metrics

Physiological metrics have several important limitations when it comes to validity, the most crucial being that they are associated with other constructs, such as workload and stress, and remain only a proxy measure of SA (Ofte & Katsikas, 2023).

Two systematic reviews included in this meta-review focused on eye tracking metrics for SA (Arias-Portela et al., 2024; Zhang et al., 2023). Arias-Portela et al. (2024) looked at the use of eye tracking within the driving domain, whereas Zhang et al. (2023) considered physiological metric-based measures of SA across domains. Arias-Portela et al. (2024) found that especially fixations, pupil diameter, and saccades were associated with SA in the included primary studies. Distracted drivers had higher fixation durations and lower fixation counts on areas of interest, and the more dispersed the visual attention of the drivers, the greater their SA. However, they did not state with what measure of SA they compared the eye-tracking metrics.

Zhang et al. (2023) found positive correlations between direct measures of SA and conscious eye tracking metrics, such as fixation rate and count and dwell time, but not between direct measures of SA and unconscious metrics, such as blink rate and pupil dilation. Their review included primary studies that looked at various direct SA measures: Ten of the included studies employed SAGAT (or a variant), and four of these found significant correlations between at least one eye-tracking metric and SAGAT scores; five studies used SART, and three of them found significant correlations; two studies used CARS, and one found a significant correlation. In primary studies where no correlation was found between eye-tracking metrics and direct measures of SA, researchers reported nonetheless a positive association between eye-tracking metrics and performance ratings, and only a much weaker association between the direct SA measure and performance ratings. This suggests that eye-tracking metrics may have positive predictive validity.

Despite the positive convergent and predictive validity evidence, eye tracking was sensitive to cognitive workload, emotion, study design, and task (Arias-Portela et al., 2024; Zhang et al., 2023). Finally, research highlighted that eye tracking only captures level 1 SA (Arias-Portela et al., 2024; Cheng & Esmaeili, 2024; Tan & Zhang, 2024), negatively affecting its content validity. Other physiological measures, such as EEG, may be used to offer insights into brain activity and cognitive processes related to level 2 and 3 SA (Tan & Zhang, 2024).

Zhang et al. (2023) also presented validity evidence concerning the use of cardiovascular metrics as an indicator of SA. They found six papers in which cardiovascular metrics and other measures of SA were both used. Three of six studies used self-ratings, and correlations between cardiovascular metrics and self-rated SA were observed in two out of three of these studies. The other three studies used SAGAT, and two out of three of these observed a positive correlation between average heart rate and SAGAT scores. Three studies reported by Zhang et al. (2023) examined the impact of display design on heart rate, two of which found higher heart rates for displays associated with higher SA. As for eye tracking metrics, cardiovascular metrics have also been associated with workload. Additionally, cardiovascular metrics were influenced by task duration, as shorter tasks did not allow sufficient time for cardiovascular responses to stabilise.

Finally, Zhang et al. (2023) reviewed three studies using EEG, all of which reported positive correlations with SA scores obtained using probing techniques such as SPAM, SAGAT, and QASA. One study simulated a loss of SA and was able to measure an impact on brain activity.

#### Reliability of Physiological Metrics

No reliability indices were reported in the systematic reviews of physiological metrics-based measures of SA. Arias-Portela et al. (2024) did present a study showing that eye-tracking metrics were less reliable outside of laboratory settings because of variable light conditions. In addition to task and environment characteristics such as illumination intensity, eye-tracking metrics were also sensitive to individual characteristics (e.g., age and pupil colour).

#### Usability of Physiological Metrics

Physiological metrics were praised for providing non-intrusive measures that integrate seamlessly into the operational flow (Tan & Zhang, 2024). Nevertheless, various usability issues were mentioned in the systematic reviews, mostly due to the current technological limitations of physiological metrics. For example, Cheng and Esmaeili (2024) stated that, while eye tracking is a non-intrusive technique, it is impractical to implement because it is not comfortable for people with glasses. Furthermore, because of their sensitivity to the environment, physiological metrics require a controlled environment (Arias-Portela et al., 2024). However, there are already some solutions for mitigating environmental factors, such as eye illuminators (Arias-Portela et al., 2024). Since physiological metrics can also be affected by individual characteristics, they do require individual calibration (Arias-Portela et al., 2024; Zhang et al., 2023).

### Other Measures

Another approach to SA measurement reported in various reviews involved using performance as a proxy for SA (Meireles et al., 2018; Ofte & Katsikas, 2023; Orique & Despins, 2018; Tan & Zhang, 2024). However, this method rests on the erroneous assumption of a direct causal relationship between SA and performance (Meireles et al., 2018; Tan & Zhang, 2024), overlooking potential moderating and mediating factors. In addition to performance-based measures, several other alternative tools (e.g., Random Number Cognition Test, Laparoscopic Cholecystectomy Postprocedural Assessment, and Checklist Perception Survey) and techniques (e.g., verbal protocol analysis, task analysis, video studies, focus groups, open-ended question, closed-ended questions, and interviews) were each mentioned in one of the included reviews, apart from interviews, which were mentioned in three reviews. However, these had to be excluded from our analysis, as they were not described sufficiently to be identified and categorised as SA measurement tools.

### Critical Appraisal

The critical appraisal resulted in a mean CASP score of 5.4 out of 7 (SD = 1.2; range = 2–7) for the included reviews. With the exception of two reviews (Arias-Portela et al., 2024; Cooper et al., 2013), all reviews scored 5 or higher. CASP scores were typically reduced for two main reasons. First, several reviews failed to provide sufficient information about their search strategy. Second, many reviews either did not assess the quality of the included primary studies or did not clearly report the results of this assessment. Only four reviews explicitly reported on conducting a quality assessment of included studies, and only three clearly presented the results of this assessment. The quality of the primary studies evaluated in these three reviews varied substantially (Ghaderi et al., 2023; Orique & Despins, 2018; Zhang et al., 2023). Table A1 in the supplementary material provides an overview of CASP scores for each review, along with details of the quality assessments of primary studies where these were conducted and reported.

### Cross-Review Comparison

Cross-review comparison is somewhat limited because there was little overlap between the included systematic reviews. The reviews covered different domains or tools, did not all present psychometric evidence, and, when such evidence was reported, often focused on different psychometric properties. Nevertheless, we found enough material on observer ratings, SAGAT, and eye-tracking metrics to make a comparison across reviews.

#### Observer ratings

Three reviews focussing on the healthcare domain covered observer ratings and presented validity and reliability evidence (i.e., Cooper et al., 2013; Ghaderi et al., 2023; Orique & Despins, 2018). The reviews were published five years after one another. Ghaderi et al. (2023) and Orique and Despins (2018) had only five primary studies in common (out of 49 unique primary studies), but their results were well aligned. Cooper et al. (2013) did not clearly report the primary studies included, which was also reflected in its lower CASP score. Accordingly, our conclusions place greater weight on the findings reported by Ghaderi et al. (2023) and Orique and Despins (2018) when evaluating the evidence for observer ratings in healthcare.

#### SAGAT

All reviews, except for Arias-Portela et al. (2024), discussed the use of SAGAT and provided complementary psychometric evidence. However, some disagreement emerged regarding the intrusiveness of SAGAT, likely stemming from varying interpretations of what constitutes intrusiveness. Specifically, some reviews focused on SAGAT’s impact on performance and workload, while others emphasised its potential effects on SA and disruptions to safety-critical workflows in real-life settings.

#### Eye-tracking metrics

Both Arias-Portela et al. (2024) and Zhang et al. (2023) reviewed the psychometric properties of eye-tracking metrics, with largely complementary findings. However, a difference emerged in their conclusions regarding the validity of unconscious metrics, particularly pupil dilation. While Arias-Portela et al. (2024) found an association between pupil diameter and SA, Zhang et al. (2023) reported no significant correlation between unconscious eye metrics and a direct measure of SA. These reviews, published in close succession and sharing only one overlapping primary study, offer valuable insights into the role of eye tracking in different contexts. While Arias-Portela et al. (2024) specifically focused on drivers, Zhang et al. (2023) took a broader approach, not tied to a particular domain. When interpreting the evidence across both reviews, it is important to consider that, based on our CASP appraisal, Zhang et al. (2023) provided a more rigorous evaluation of primary study quality and more transparent and comprehensive reporting of the search strategy, interpretation of results, and outcomes.

## Discussion

While systematic reviews on SA measurement have focused on specific measurement tools, domains, or psychometric properties, this meta-review synthesised the evidence on the characteristics and psychometric properties of SA measures across domains and tools. Based on 14 systematic reviews covering more than 477 unique primary studies, we identified 38 SA measurement tools and categorised them as self-ratings, observer ratings, probing techniques, or physiological metrics. Overall, probing techniques, especially the Situation Awareness Global Assessment Technique (SAGAT), yielded the most substantial and strongest validity evidence but were also most criticised for their usability. Our synthesis of data across domains identified the complementary strengths of SA measurement tools and enables the identification of best practice recommendations for practitioners and researchers aiming to measure or study SA in any context.

### Situation Awareness Measures Across Contexts

In addition to providing a comprehensive overview of SA measurement tools, this meta-review considered the specific contexts in which the tools were used, that is, study populations, units of measurement (individuals, groups, and systems), and settings (both simulation and real-life), thereby contributing to a better understanding of how the context influences the selection of appropriate SA measures.

#### Populations

While there were no differences in the use of most SA measurement tools across domains, research in healthcare professionals uniquely employed NTS observer ratings to assess SA. This finding underscores that SA in healthcare is often assessed in conjunction with other NTS, viewed as a skill rather than a cognitive state, and evaluated through observable behaviours (Chandanani et al., 2025). Nevertheless, much of the research on SA measurement in healthcare professionals remains grounded in a positivist, cognitive engineering tradition, which does not always capture the socio-cultural and distributed nature of healthcare teams (Walshe et al., 2021). This aligns with Meireles et al.'s (2018) review of the conceptualisation of SA across operational domains, which showed that while similar definitions of SA are used across domains, the operationalisation of SA varies, with some domains emphasising cognitive processes and others focussing on observable behaviours. These different operationalisations reflect significant differences in domain characteristics such as training rigour, organisational structures, and cultural practices (Gaba, 2000; Kapur et al., 2016).

Exploring the operationalisation of SA in emerging fields may hold potential for cross-domain innovation and learning. For example, fields like cybersecurity, characterised by dynamic and less tangible environments, challenge conventional SA models as these may not fully capture the domain’s complexities (Meireles et al., 2018). This offers opportunities to further our understanding of what SA is and how to best measure it.

#### Units of Measurement

The diversity of tools and approaches applied across units of measurement highlighted the complexity of measuring SA and the unique challenges posed by the measurement of individuals, groups, and systems. While observer ratings were more common for group-level measurement, which makes sense given their heavy reliance on observable coordination between team members, probing techniques were suitable for measuring SA in both individuals and groups of individuals. Few tools were used to measure system SA.

According to our review, system SA has been measured in cybersecurity using self-ratings and proxy measures such as eye-tracking metrics (Ofte & Katsikas, 2023). However, a closer look at the primary studies reveals that, although many articles highlighted the importance of system SA, few described specific tools for its measurement. Where measurements were explicitly discussed, they typically focused on modelling of SA rather than direct measurement. For example, Dutt and Gonzalez (2012) developed a cognitive instance-based learning model of cyber security in a cyber-attack scenario, in which predictions about recognition and comprehension processes were generated and evaluated using two cyber-SA metrics: accuracy and timeliness. Salmon, Stanton, Walker, Jenkins (2009) described several case studies conducted in both civilian and military complex collaborative environments in which they aimed to validate the propositional network approach as a method for measuring distributed SA, mapping knowledge elements and their relationships into propositional networks. Future research should explore how modelling and other approaches can be validated and applied for system SA measurement in different contexts. 

It is important to develop measurements that take the socio-technical system as the unit of analysis rather than the cognition of individuals, because performance is not determined entirely by the information-processing properties of (the sum of) individuals (Hutchins, 1995). Instead, effective performance in complex environments emerges from patterns of coordination, communication, and adaptation within teams, with awareness dynamically distributed across people, artefacts, and contexts (Bergström et al., 2010, 2011). This might be especially relevant in domains such as healthcare, where professionals must dynamically collaborate across disciplines and levels of responsibility (Perry et al., 2006), or in cybersecurity, where systems also include non-human agents that cannot easily be questioned, observed, or physiologically tracked. Treating SA as an individual ability rather than a system-wide capability is not only theoretically limiting but also potentially dangerous. As Dekker (2015) cautions, this can lead to misuse in attributing blame, turning SA into a retrospective judgement used to accuse practitioners of ‘losing SA’ in incident investigations.

From a distributed-SA perspective, the analytical focus shifts from what the level of SA is to *how* and *why* certain conditions enable or constrain its formation: how information flows, how cues are interpreted collectively, and how shared understanding is maintained or lost under pressure. In this vein, Bergstrom et al. (2011) proposed Operational Resilience as a distributed cognition–based alternative to traditional Crew Resource Management. It shifts attention from predefined behavioural indicators and normative conceptualisations of SA toward the situated processes of coordination and shared sense-making that sustain performance in complex sociotechnical systems.

#### Settings

SA measures in all four categories have been employed in both real-life and simulation settings, but most studies were conducted in simulated environments. For instance, Arias-Portela et al. (2024) reported that only 12% of primary studies occurred in real-life settings. Similarly, in sports, most research was conducted in simulated settings (Huffman et al., 2022). Simulations provide a safe, more manageable environment for research, making their predominance unsurprising. Furthermore, in fields like sports, where professionals spend most of their time training, it is more appropriate to validate measures in simulations.

However, real-life studies are generally considered superior for validation, despite their higher costs and greater ethical, safety, and data collection requirements, because they better capture emotional responses, reactions, attention, and contextual perceptions (Arias-Portela et al., 2024). For example, Wintersberger et al. (2023) found that drivers exhibited faster reaction times in naturalistic studies than in simulations, underscoring how study settings can influence outcomes. With improved simulator fidelity, this gap between simulation and real-life settings is narrowing. Advances in extended reality and other technologies enable simulations that replicate natural experiences more closely (Herur-Raman et al., 2021) and can be employed as a practical means of allowing for more realistic SA measurement and improved validation of SA measures.

### Psychometric Properties of Situation Awareness Measures

This meta-review revealed substantial gaps in psychometric evidence for SA measurement tools, as well as considerable variation in how psychometric properties were tested and reported, making it difficult to integrate the existing evidence into a complete and coherent picture. The most reported psychometric property was agreement with other SA tools, but rigorous independent validation, such as sensitivity to SA manipulations, is lacking for most tools. Future research should employ established methodologies like COSMIN (COSMIN Taxonomy of Measurement Properties, nd) to obtain more structured and detailed evidence on the tools’ psychometric properties.

#### Validity

Probing techniques, especially SAGAT, are the most thoroughly validated category of SA measures. Generally, SAGAT has been found to demonstrate high validity when conducted as originally prescribed. Compared to real-time probes (such as SPAM), SAGAT was both more sensitive and more predictive of performance. All evidence presented in support of SAGAT’s validity comes from Endsley’s (2020, 2021) reviews, which may raise concerns about the independence of the evidence base. Bakdash et al. (2020) further critiqued the quantitative synthesis reported in Endsley (2021), warning that certain significance-filtering methods can inadvertently overestimate predictive validity. Endsley’s (2020, 2021) reviews encompass a large and diverse body of research, summarising more than 200 primary studies conducted by numerous authors across a wide range of domains, which mitigates concerns about the concentration of evidence but, at the same time, posed challenges for conducting a meta-analysis.

Questions remain about other aspects of SAGAT’s validity. For example, Sarter and Woods (1995) argued that halting a simulation and prompting participants for information may disturb the very SA the researcher aims to measure. They state that the prompts themselves can act as retrieval cues, influencing what knowledge the participants recall and how they perceive its relevance, potentially altering their natural cognitive processes. This concern of intrusiveness has partially been resolved by evidence that SAGAT does not negatively affect workload or performance (Endsley, 2021), but more research is needed to determine whether SAGAT and similar techniques alter SA.

Another concern is that SAGAT uses situation-specific probes rather than standardised questions, making its validity dependent on probe quality. While guidance exists on the sequence of steps in the Goal-Directed Task Analysis for identifying SA probes, the instructions remain largely conceptual, lacking practical clarity and creating ambiguities (Nasser-Dine, 2021). Importantly, although scoring in SAGAT is objective, the design and selection of probes require subjective expert judgement, which introduces an additional source of variability that may influence outcomes. Moreover, probes used in published studies might be of higher quality than those used in unpublished studies or in practice, giving us a potentially biased picture of the actual sensitivity and predictive validity of SAGAT.

The widespread use of SAGAT is likely influenced by its close alignment with Endsley’s (1995b) widely accepted three-level model of SA that has guided much of the field’s theoretical development. This alignment also helps to explain why SAGAT yielded more positive validity evidence compared to other tools. Other tools are often validated against SAGAT or with reference to its (cognitive) three-level theoretical foundation, even when they may measure different aspects of SA.

Self-ratings, observer ratings, and physiological measures offered valuable insights into other relevant aspects of SA. Findings suggest that self-ratings primarily assess an individual’s confidence in SA rather than objective SA, and the calibration of this confidence is important for performance. For example, Sethumadhavan (2011) explains that individuals must have accurate metacognitive judgements about their SA to adopt better monitoring strategies and effectively respond to automation failures. Observer ratings reflect observable behaviours relevant to establishing and maintaining SA. Positive evidence for the content validity of observer ratings, alongside inconsistent evidence for their structural and criterion validity, suggests that observer ratings may be measuring different constructs, including teamwork processes and communication patterns, which are nonetheless critical for SA. Physiological metrics offer objective measures of processes related to SA and other performance-critical cognitive states, such as stress and workload, making them valuable indicators for tracking temporal dynamics.

Although self-ratings, observer ratings, and physiological metrics capture relevant aspects of SA, they do not fully align with Endsley's (1995b) definition of SA. While this misalignment may be perceived as undermining their validity, it highlights the multi-faceted and contested nature of SA and underscores the need for conceptualisations that extend beyond a purely cognitive framework.

#### Reliability

Most of the available evidence for reliability of SA measures was found for observer ratings. Evidence for the reliability of observer ratings was drawn from four reviews and varied considerably between tools. Interestingly, our review showed that the reliability of the SA component in NTS observer ratings was often lower than that of the other NTS components. Using observation, SA may be more difficult to assess than other NTS, such as communication, because it cannot be directly observed. Similarly, we found challenges to the reliability of physiological metrics. Physiological metrics can be sensitive to the experimental environment, as well as the characteristics of the subject, and thus depend on the controlled nature of the study environment, calibration procedures, and the technological advancement of the tools used. 

Recent advancements, such as the integration of EEG and eye-tracking data (Nolte et al., 2024), and the development of guidelines for optimising eye-tracking setups to mitigate environmental and user-specific variability (Molina et al., 2024), highlight ongoing efforts to enhance the reliability and applicability of physiological metrics-based tools. Moreover, advanced machine learning models or augmented reality interfaces could facilitate real-time SA assessment (Tan & Zhang, 2024), and communication and movement-based analysis could become more viable as automatic speech recognition and AI-assisted behaviour-tracking technology advances (e.g., Kwok & Virdi, 2022).

Beyond measurement, advanced technologies also enhance SA data evaluation and interpretation, as seen in computer vision applications that use team motion metrics to assess SA in complex environments (Dias et al., 2022). Currently, these technologies remain highly experimental and are therefore far from routine use. Future research on integrating physiological metrics with advanced interpretation models could revolutionise SA measurement accuracy and efficiency.

#### Usability

Evidence on the usability of SA measures is limited, with most research focussing on the potential intrusiveness of SAGAT and the ease of use of self-ratings. A key concern with SAGAT is its requirement to freeze a scenario at multiple random points, which can disrupt the natural flow of tasks. Endsley (2021) and Orique and Despins (2018) demonstrated that these interruptions do not negatively affect workload or performance. Endsley (2011) highlights that this applies not only to objective performance indicators but also to subjective performance. Despite this evidence suggesting that SAGAT’s intrusiveness may be limited in controlled studies, its application remains impractical in safety-critical, real-life settings where tasks cannot be paused. Even in scenarios where freezes do not pose a safety concern, some studies have reported participant critiques on the disruptions caused by SAGAT freezes (e.g., Strybel et al., 2008). Thus, while our review found evidence that SAGAT does not affect performance, the subjective experience of disruptiveness could still influence the acceptance of the measurement tool. When freezing a task is not an option, real-time probes such as SPAM may offer a feasible alternative for assessing SA according to Salmon et al. (2006). However, they acknowledge that receiving and responding to queries still introduces some level of disruption to the primary task.

In contrast, our review showed that self-ratings are easy to use because they do not require external observers like observer ratings, freezes and context-specific queries like probing techniques, or costly equipment, calibration, and data processing like physiological metrics. However, these advantages also introduce limitations for self-ratings. For example, while SART questions are broadly applicable across domains and roles, their lack of task specificity reduces their ability to provide detailed diagnostic insights into the factors contributing to adequate SA (Strybel et al., 2007). Additionally, self-ratings only require one post-hoc administration, meaning they cannot capture dynamic changes in SA throughout tasks.

Overall, our findings reveal a lack of attention to the usability of SA measurement tools. The Human Factors community should support practitioners by developing accessible, validated tools rather than compromising on usability. Future research should focus on creating new tools that balance validity and reliability with usability, as well as on improving the usability of existing tools. For example, full reporting and broader dissemination of well-designed SA probes would improve SAGAT’s feasibility. Furthermore, there are existing scales and guidelines that could be adapted and used to assess the usability of SA measurement tools, one such example being the System Usability Scale (Brooke, 1996). As Brooke (1996) highlights, the usability of a tool depends on the tool’s appropriateness to the context in which it is used, as reflected, for example, in our findings that some measurement tools may be more practical in simulations than in real-life settings. Thus, the usability of SA measurement tools should be assessed within the context of their intended purpose.

### Practical Implications

This meta-review confirms that SAGAT, a probing technique, is the current gold standard for SA measurement, with substantial evidence supporting its validity across a wide range of domains. When using SAGAT, however, it is essential to follow recommended procedures and acknowledge its limitations (Endsley, 2021; Orique & Despins, 2018). SAGAT has often been adapted for practical reasons, potentially reducing its validity. Therefore, we stress that researchers and practitioners adhere to published SAGAT protocols, including to avoid freezes too early in a trial (i.e., not before 3 minutes), to space freezes 3 to 6 minutes apart, to schedule freezes randomly to prevent anticipation, to use at least two to three freezes per scenario rather than too few or only end-of-trial probes, to keep freeze durations brief (≤2 min), to provide three to five training trials before testing, to draw on a wide range of queries covering perception, comprehension, and projection, to avoid the combination of scores across freezes or SA levels, to use approximately 10–15 queries per freeze, and to ensure that displays or other relevant cues are hidden during freezes (Endsley, 1995a, 2021; Salmon, Stanton, Walker, Jenkins, et al., 2009). If adaptations are unavoidable, they should be clearly documented, and their impact on the validity and reliability should be carefully assessed. Because SAGAT probes are not standardised as in validated questionnaires, we further recommend that researchers always publish the probes they used to enhance transparency and to facilitate interpretability and replication.

Although SAGAT represents the most extensively validated SA tool, it may not always be the most suitable choice. Selecting an appropriate SA measure requires aligning the tool with (a) the purpose of the assessment (e.g., research, training, or operational improvement), (b) the conceptualisation of SA being applied (e.g., the three-level model of SA), and (c) practical constraints (e.g., available resources and characteristics of the target population). To support this process, Table 4 summarises the practical guidance for selecting SA measurement tools derived from our review. More specifically, it presents a structured three-step process built around guiding questions that form part of our proposed tool selection framework: first, defining the purpose of the measurement; second, identifying the corresponding conceptualisation of SA. Together, these two steps inform which measurement options are appropriate, while the third step considers practical constraints to further narrow the range of suitable options. Below, we explain the logic of these steps in more detail.

**Table 4.** Practical Guidance for Selecting Situation Awareness Measurement Tools.

Steps	Questions	Options	Recommendations
1. Establish Purpose of Measurement	What is the purpose of the measurement?	Examples:	
		1. To train and improve SA in individuals or groups (e.g., simulation training)	1. Consider SA as a skill; focus on behavioural or metacognitive (group) dimensions
		2. To evaluate the impact of new interfaces, procedures, or automation on SA	2. Consider SA as a momentary state; focus on cognitive or behavioural dimensions
		3. To research the link between SA fluctuations with workload, tasks, or performance	3. Consider SA as a dynamic momentary state; focus on cognitive dimensions with temporal sensitivity
2. Define Conceptualisation	How do I conceptualise SA?	[ ] Momentary state	Use self-ratings, probing techniques, and/or physiological metrics
		[ ] Trainable skill	Use self-ratings, observer ratings, and/or probing techniques
	Which dimension of SA is my focus?	[ ] Behavioural	Use probing techniques and/or physiological metrics
		[ ] Metacognitive	Use self-ratings
		[ ] Cognitive	Use observer ratings
	What is the locus of the SA?	[ ] Individual	Use self-ratings, observer ratings, probing techniques, and/or physiological metrics
		[ ] Group	Use team self-ratings, team observer ratings, team probing techniques, and/or physiological metrics
		[ ] System	At present, no SA measurement tools can be recommended for assessing system-SA
	What type of output do I want from the measurement?	[ ] Absolute score – provides a numeric score anchored to a scale	Use self-rating, observer rating, and/or probing techniques
		[ ] Relative measure – shows directional or proportional patterns without a natural benchmark or reference point	Use self-rating, observer rating, probing techniques, and/or physiological metrics
		[ ] Diagnostic insight – yields explanatory information about why SA was high or low	Use observer ratings and/or probing techniques
	What temporal resolution of SA is my focus?	[ ] Dynamic (temporal fluctuations)	Use physiological metrics
		[ ] Intermittent (sampled points)	Use observer ratings, probing techniques, and/or physiological metrics
		[ ] Static (overall snapshot)	Use self-ratings, observer ratings, probing techniques, and/or physiological metrics
3. Consider Practical Constraints	Can the task be interrupted?	[ ] Yes	No → Probing techniques cannot be used
		[ ] No	
	Are subject matter experts available to design and/or conduct the measurement?	[ ] Yes	No → Probing techniques and/or observer ratings cannot be used
		[ ] No	
	Is there observable behaviour or communication to rate?	[ ] Yes	No → Observer ratings cannot be used
		[ ] No	
	Can subjects be observed directly (live) or indirectly (video/audio recordings)?	[ ] Yes	No → Observer ratings cannot be used
		[ ] No	
	Can subjects be asked to answer questions?	[ ] Yes	No → Self-ratings and probing techniques cannot be used
		[ ] No	
	Can subjects wear and calibrate devices?	[ ] Yes	No → Physiological metrics cannot be used
		[ ] No	
	Is there access to technological equipment and expertise?	[ ] Yes	No → Physiological metrics cannot be used
		[ ] No	

*Note*. SA = situation awareness.

#### The ‘Why’ and the ‘What’

The purpose of the assessment (the ‘why’) should guide the conceptualisation of SA (‘what’ exactly is to be measured). For instance, training SA skills requires more than knowing how ‘low’ or how ‘high’ SA was at a given time; it requires identifying areas of improvement and thus a focus on skills that can be observed and trained rather than unconscious cognitive or physiological processes.

Researchers and practitioners who wish to assess SA must recognise that the measurement of SA is never theory neutral, meaning that you cannot measure SA without making assumptions about what SA actually is. Every way of measuring SA inevitably reflects a particular theoretical stance on the nature of SA, and the validity of any measure depends on its alignment with its conceptualisation. As shown throughout this review, there are multiple ways to operationalise SA, each corresponding to a different theoretical perspective. Therefore, we recommend making explicit which conceptualisation of SA is being used and then identify a type of SA measure that matches that stance.

Our review showed how different measures map onto different theoretical perspectives: probing techniques, especially SAGAT, align closely with the three-level model of SA (Endsley, 1995b) and capture SA as a momentary cognitive state; self-ratings tap into metacognitive judgement; observer ratings focus on behavioural manifestations and skills related to establishing and maintaining SA; and physiological metrics such as eye tracking highlight the temporal dynamics of attentional allocation. Considering tools other than SAGAT therefore inherently implies an alternative framing of SA, such as a behavioural rather than a purely cognitive perspective when using observer rating methods. Likewise, a systems approach to SA requires an operationalisation that captures interactions between system components, such as the propositional network approach, rather than individual metacognitive measurement through SART. Advancing SA measurement thus requires progress on theory and method in concert.

#### Practical Constraints

Once the ‘why’ (purpose of assessment) and the ‘what’ (conceptualisation) have been established, the next step is to operationalise the chosen conceptualisation into a concrete measurement approach (the ‘how’). Here, practical constraints must be considered. For example, in settings where tasks cannot be interrupted without compromising safety, SAGAT cannot be administered. But this constraint does not necessarily pose a problem in simulations, where freezes can be scheduled without safety implications. Similarly, when a participant performs a task individually and without communication, observer ratings are not feasible. In situations where analysis is limited to recordings and direct access to participants is not possible, observer ratings, unlike other types of measures, can be highly practical.

This structured process of reflecting on the underlying ‘why’, ‘what’, as well as practical constraints within a given operational context, and their implications for SA measurement, supports researchers and practitioners in making grounded, context-appropriate choices regarding tool selection.

### Strengths and Limitations

When interpreting the findings of this meta-review, it is important to consider its strengths and limitations. First, many included systematic reviews lacked sufficient detail about how SA measurement outcomes were interpreted in the primary studies, as well as about the specific contexts in which individual pieces of psychometric evidence were obtained. Consequently, our conclusions regarding psychometric properties reflect the general characteristics of SA measurement tools rather than their application in specific circumstances. At the same time, this relatively broad perspective represents a strength of the review, as it allows the identification of patterns in the evidence base for SA measurement tools.

We selected a meta-review approach because the SA measurement literature is extensive and heterogeneous. The spread of the literature across different domains, and across multiple reviews, has to-date stymied learning from the totality of the work considering SA measurement and complicated decision making for both researchers and practitioners. In such instances, meta-reviews serve an important clarifying function by integrating disparate findings into a coherent evidence base (Ioannidis, 2016). Although meta-reviews represent a relatively recent development in review methodology, they are now well-established and guided by recognised reporting standards, such as the PRIOR statement (Gates et al., 2022) which we followed in this work. Adopting this approach enabled us to synthesise findings from the broad and diverse evidence base, highlight consistencies and contradictions, and distil practical recommendations and research gaps without oversimplifying the literature. Our synthesis aggregated evidence from more than 477 unique primary studies, with only 47 overlapping across reviews, underscoring the value of a meta-review in integrating dispersed findings into a comprehensive perspective on SA measurement.

At the same time, we set a clear scope by focussing specifically on reviews of SA measurement. This meant that we excluded reviews on NTS that did not specifically address SA, but included those primarily focused on SA, even if they covered NTS. As this still yielded many NTS measurement tools, we believe our approach did not unduly bias the results. By focussing solely on SA, we also excluded research on related constructs referred to by different terms, such as *vigilance* in neuroscience (Sebastiani et al., 2020). The existence of overlapping constructs described using diverse terminology highlights the complexity of SA conceptualisation and the difficulty of capturing its multifaceted nature across disciplines.

Finally, although the systematic and rigorous meta-review approach adopted in this study is a key strength, the overall quality of any meta-review ultimately depends on the quality of the systematic reviews it includes. Our critical appraisal indicated that most reviews achieved relatively high CASP scores, but many lacked sufficient detail regarding their search strategies and did not conduct or report a quality assessment of the primary studies. Among the few reviews that did assess the quality of primary studies, reported levels of quality varied considerably. These findings underscore the need for future reviews to apply robust appraisal frameworks and for primary research to adhere to higher methodological and reporting standards in order to strengthen the evidence base on SA measurement.

### Future Research Directions

This meta-review highlighted substantial gaps in the psychometric evidence of SA measurement tools. These gaps differ by measurement category but converge on the need for more systematic and rigorous research using structured frameworks such as COSMIN to standardise how the psychometric properties of SA tools are tested and reported. We mapped our results to the COSMIN framework to identify specific gaps in the body of evidence.

For self-ratings, mapping of results to the COSMIN framework shows a lack of strong evidence for content validity, internal consistency and test–retest reliability, and a lack of evidence for structural validity, predictive validity, criterion validity, measurement error, and sensitivity. To advance this category of SA measures, future research should systematically evaluate self-rating tools, especially the widely used SART, across these COSMIN properties.

For observer ratings, COSMIN mapping of the results indicates a lack of consistent evidence for content validity, internal consistency and interrater reliability; a lack of strong evidence for structural, construct, and criterion validity; and a lack of evidence for test–retest reliability, measurement error, and sensitivity. Future work should (i) move beyond interrater indices to evaluate structural validity and establish stronger external benchmarks for criterion validity and (ii) investigate why reliability and content validity vary across tools and why reliability of the SA component in NTS observer ratings often scores lower than that of the other NTS components.

For probing techniques, COSMIN mapping of the results revealed a lack of strong evidence for content validity; a lack of evidence for structural validity and measurement error; and a lack of consistent evidence for reliability. Most of the available psychometric evidence related to SAGAT, with very little evidence reported for variants of this technique. Beyond COSMIN, concerns remain about potential intrusiveness of freezes. Future research should (i) strengthen the evidence base for SAGAT on COSMIN properties where evidence is weak, inconsistent, or absent; (ii) address the paucity of evidence for SAGAT variants; (iii) establish transparent and replicable procedures for the development of valid probes; and (iv) clarify whether, and under what conditions, probing intrudes on SA itself.

For physiological metrics, COSMIN mapping of the results showed a lack of evidence for structural validity, measurement error, and sensitivity; and a lack of strong evidence for convergent, predictive, and criterion validity, as well as for reliability. Most of the available evidence stems from eye-tracking metrics. Future research should (i) expand beyond eye tracking to other physiological measures; (ii) build evidence across COSMIN properties where it is weak, inconsistent, or lacking; and (iii) test recent technological advances for their psychometric performance, establishing whether and how they improve validity, reliability, and usability.

At a more general level, several priorities for the field of SA measurement emerged from this review. One important research direction is to investigate alternative conceptual frameworks beyond the three-level model of SA by Endsley (1995b), particularly to assess the validity of self-ratings, observer ratings, and physiological metrics. Additionally, it would be interesting to explore how SA is operationalised in emerging fields that challenge conventional cognitive models to drive theoretical innovation.

Methodologically, greater emphasis is needed on testing tool sensitivity to SA manipulations and on conducting independent validation, rather than relying primarily on tool-to-tool agreement. Future validation efforts should also incorporate systematic usability assessments to ensure that tools are practical and effective in their intended contexts. Another important direction for future research is to develop and validate dedicated tools for capturing system-level SA. Finally, as the evidence base of new and existing tools continues to grow, future reviews will play an important role. To maximise their value, they should include robust critical appraisal of primary studies to improve the quality and interpretability of evidence syntheses.

## Conclusion

This meta-review synthesised evidence on the characteristics and psychometric properties of SA measurement tools across all human factors domains. Probing techniques, particularly the Situation Awareness Global Assessment Technique (SAGAT), demonstrated the strongest validity evidence but raised usability concerns. While probing techniques are well supported, they are not always the most suitable choice, and other categories of measures can provide complementary benefits: Self-ratings to capture metacognitive judgements of SA, observer ratings to assess behavioural manifestations and skills involved in establishing and maintaining SA, and physiological metrics to track the temporal dynamics of processes related to SA. This underscores the importance of aligning SA measurement tools with the purpose of use and the corresponding conceptualisation of SA. Looking ahead, theoretical, methodological, and technological advances offer promising opportunities to refine SA measurement, ultimately enhancing our understanding of the factors influencing SA and providing better support to professionals across domains in establishing and maintaining SA.

## Key Points

• A review of systematic reviews across domains identified 38 SA measurement tools categorised as self-ratings, observer ratings, probing techniques, and physiological metrics.
• Probing techniques best aligned with the most widely adopted definition of SA and demonstrated the strongest validity evidence but were not practical for all measurement purposes and contexts of use.
• Self-ratings, observer ratings, and physiological metrics provided complementary benefits for specific purposes and contexts of use but require different conceptualisations of SA.

## Supplemental Material

Supplemental Material - Methods and Skills Measuring Situation Awareness: A Meta-Review Across Domains Supplemental Material for Methods and Skills Measuring Situation Awareness: A Meta-Review Across Domains by Laura Louise Moens, Sinéad Lydon, Sara Cucurachi, Paul O’Connor, Thomas Christian Sauter, Gian-Andri Töndury, Tanja Manser in Human Factors
