# Supplementary material for: Measuring Situation Awareness: A Meta-Review Across Domains
Source: Hum Factors. 2026 Jan 17;68(5):632–72. doi: 10.1177/00187208251412110 (PMC13013663; doi:10.1177/00187208251412110)
Supplement: Supplemental Material - Methods and Skills Measuring Situation Awareness: A Meta-Review Across Domains [file sj-pdf-1-hfs-10.1177_00187208251412110.pdf]

MEASURING SITUATION AWARENESS

Supplementary Material 1. Example Search Strategy for MEDLINE (Ovid)

Search terms for situation awareness:

1. situation* adj1 aware*.ab,ti.
2. SAGAT.ab,ti.
3. SPAM.ab,ti.
4. SALSA.ab,ti.
5. SAVANT.ab,ti.
6. QUASA.ab,ti.
7. SACRI.ab,ti.
8. SART.ab,ti.
9. Awareness/
10. 1 OR 2 OR 3 OR 4 OR 5 OR 6 OR 7 OR 8 OR 9

Search terms for measurement:

11. Measure*.ab,ti.
12. Assess*.ab,ti.
13. Metric*.ab,ti.
14. Examin*.ab,ti.
15. Evaluat*ab,ti.
16. SAGAT.ab,ti.
17. SPAM.ab,ti.
18. SALSA.ab,ti.
19. SAVANT.ab,ti.
20. QUASA.ab,ti.
21. SACRI.ab,ti.

22. SART.ab,ti.
23. survey*1.ab,ti.
24. questionnaire*1.ab,ti.
25. instrument*1.ab,ti.
26. tool*1.ab,ti
27. Rate*1.ab,ti.
28. Rating*1.ab,ti.
29. Technique*.ab,ti.
30. Analy*.ab,ti.
31. Inventor*.ab,ti.
32. Scale*.ab,ti.
33. 11 OR 12 OR 13 OR 14 OR 15 OR 16 OR 17 OR 18 OR 19 OR 20 OR 21 OR 22 OR 23 OR 24 OR 25 OR 26 OR 27 OR 28 OR 29 OR 30 OR 31 OR 32

Search terms for systematic review

34. Meta adj1 analys*.ab,ti,kf.
35. metaanalys*.ab,ti,kf.
36. Systematic* adj1 Review*.ab,ti,kf.
37. Literature adj1 Review*.ab,ti,kf.
38. research adj1 synthes*.ab,ti,kf.
39. evidence adj1 synthes*.ab,ti,kf.
40. metaethnograph*.ab,ti,kf.
41. meta adj1 ethnograph*.ab,ti,kf.
42. Metasummar*.ab,ti,kf.
43. meta adj1 summar*.ab,ti,kf.
44. metasynthes*.ab,ti,kf.

45. meta adj1 synthes*.ab,ti,kf.
46. critical* adj1 review*.ab,ti,kf.
47. Meta-analysis/
48. Systematic Review/
49. data ADJ1 extract*.ti,ab,kf.
50. “review”.ti
51. “review”.pt.
52. PRISMA.ab.
53. preferred ADJ1 Reporting
54. review*.ab
55. 50 OR 51
56. 49 AND 55
57. 52 OR 53
58. 54 AND 57
59. 34 OR 35 OR 36 OR 37 OR 38 OR 39 OR 40 OR 41 OR 42 OR 43 OR 44 OR 45 OR 46 OR 47 OR 48 OR 56 OR 58
60. 10 AND 33 AND 59

Supplementary Material 2. Quality assessment table

Table A1

Quality Assessment of Systematic Reviews and Primary Studies

	**Systematic Reviews[a]**								**Primary Studies[b]**	
	1. Did the review address a clearly focused question?	2. Did the authors look for the right type of papers?	3. Were all the important, relevant studies included?	4. Did the review's authors do enough to assess quality of the included studies?	5. If the results of the review have been combined, was it reasonable to do so?	6. Is it clear what are the overall results of the review?	7. Were all important outcomes considered?	**CASP Score**	Quality Assessment	Quality Score
Arias-Portela et al. (2024)	Y	Y	N	N	?	N	N	**2**	Not Reported	Not Reported
Avalos et al. (2021)	Y	Y	Y	N	Y	Y	Y	**6**	Not Reported	Not Reported
Cheng & Esmaeili (2024)	Y	Y	N	N	Y	Y	Y	**5**	Not Reported	Not Reported
Cooper et al. (2013)	Y	?	N	?	Y	Y	Y	**4**	Critical Appraisal Skills Programme (CASP) guidelines and Oxford Evidence Levels	Not Reported

Endsley (2020)	Y	Y	?	N	Y	Y	Y	**5**	Not Reported	Not Reported
Endsley (2021)	Y	Y	?	N	Y	Y	Y	**5**	Not Reported	Not Reported
Ghaderi et al. (2023)	Y	Y	Y	Y	Y	Y	Y	**7**	COSMIN Risk of Bias checklist	Low to moderate quality overall; downgraded due to risk of bias, indirectness, and imprecision.
Huffman et al. (2022)	Y	Y	Y	N	Y	Y	Y	**6**	Not Reported	Not Reported
Meireles et al. (2018)	Y	Y	Y	N	Y	Y	Y	**6**	Not Reported	Not Reported
Ofte & Katsikas (2023)	Y	Y	Y	N	Y	Y	Y	**6**	Not Reported	Not Reported
Orique & Despins (2018)	Y	Y	Y	Y	Y	Y	Y	**7**	13-item checklist developed by Desborough et al. (2011)	In all but one study, aims, objectives, methods, ethics, analyses, results, discussion, and implications were clearly described. Variability existed in reporting of sample description, statistical power, piloting, and limitations; 13 studies lacked adequate sample description; 27 did not discuss statistical power; only 8 reported piloting; 5 did not address study limitations.
Priambodo et al. (2022)	Y	Y	?	N	Y	Y	Y	**5**	Not Reported	Not Reported
Tan & Zhang (2024)	Y	Y	N	N	Y	Y	Y	**5**	Not Reported	Not Reported
Zhang et al. (2023)	Y	Y	Y	Y	Y	Y	Y	**7**	Quality assessment tool by Hawker et al. (2002)	Quality ratings varied substantially, ranging from 16 to 34 out of a maximum score of 36 (higher scores indicate better quality): 32, 16, 18, 18, 32, 30, 30, 31, 28, 26, 27, 27, 34, 31, 20, 28, 19, 24, 19, 25, 25, 33, 34, 25, and 17.

Note. [a]Assessed using the Critical Appraisal Skills Programme (CASP) tool for systematic reviews (*CASP Checklists - Critical Appraisal Skills Programme*, n.d.).

Response options are “Yes” (Y = 1 point), “No” (N = 0 points), or “Can’t Tell” (? = 0 points). [b]Extracted from the systematic reviews.
